# Multi-Cutting Improves Forage Yield and Nutritional Value and Maintains the Soil Nutrient Balance in a Rainfed Agroecosystem

**DOI:** 10.3389/fpls.2022.825117

**Published:** 2022-03-01

**Authors:** Tengfei Li, Luxi Peng, Hua Wang, Yu Zhang, Yingxin Wang, Yunxiang Cheng, Fujiang Hou

**Affiliations:** ^1^College of Pastoral Agriculture Science and Technology, Lanzhou University, Lanzhou, China; ^2^College of Ecology and Environment, Inner Mongolia University, Huhhot, China

**Keywords:** cultivated pasture, simulated grazing, harvesting hay, nutritional quality, climatic factors

## Abstract

Increasing forage yield and nutritional quality under the premise of maintaining relatively stable land area and soil nutrient content is a necessary condition for the sustainable development of grassland animal husbandry. Different cutting models [simulated grazing (SG), hay harvesting (H)] of oat (*Avena sativa*), common vetch (*Vicia sativa*) and their mixture (*Avena sativa* + *Vicia sativa*) were studied on the Loess Plateau. The results show that (1) SG could increase forage yield, crude protein, and crude fat content and decrease crude ash content. In 2014, the yield of *Avena sativa* per hectare was 3,578.11 kg higher than that of H; (2) the model analysis for predicting nutritional components showed that the Crude protein (CP) and EE contents of forages in each variety (combination) showed a linear downward trend with increasing forage yield. Redundancy analysis showed that precipitation, especially in the growing season, was positively correlated with grass yield and CP content; and (3) there were significant differences in soil organic carbon, total nitrogen, NO_3_^−^-N, and NH_4_^+^-N contents for the different forage varieties (combinations) under different use modes; the values first decreased, then increased, and finally decreased. According to the comprehensive evaluation value calculated by Technique for Order Preference by Similarity to an Ideal Solution, mixed sowing was better than monoculture, and SG obtained better results than H. Overall, mixed sowing under SG can improve forage yield and nutritional quality. At the same time, precipitation regulation is the key factor affecting the production performance of rainfed cultivated grassland on the Loess Plateau.

## Introduction

Natural grassland accounts for about 40% of China’s terrestrial area and is the basis for the grazing livestock industry in the country (Kang et al., 2007). Over the last 50 years, this sector has been facing severe challenges due to the expansion of agricultural land, increasing livestock density, overgrazing, salinization, and drought (Han et al., 2008; Lehnert et al., 2016) Generally, grassland cultivation is linked to the use of various agricultural techniques (sowing, irrigation, fertilization, weed control, etc.), the artificial establishment and cultivation of herbaceous communities to provide green fodder, hay modulation, or grazing (Allen et al., 2011). The function of cultivated grassland is not only to solve the shortage of feeding forage for livestock in winter and spring, promote grassland-livestock coupling (Hou et al., 2008), but also relieve the grazing pressure from natural grasslands and restore its ecological, economic, and cultural functions (Fang et al., 2016; Liu et al., 2017).

Governments and herders are keen to develop and utilize cultivated grassland in developed countries. Cultivated grassland area accounts for 58, 69, and 80% of grassland area in Australia, New Zealand and the Netherlands, respectively (Dove et al., 2015). The total area of cultivated grassland in China is approximately 20 million hectares, which only accounts for 5% of the grassland area (Wei et al., 2018). Therefore, the development potential of cultivated grassland is crucial to sustainable agriculture. Harvesting hay is the traditional utilization of cultivated grassland (Kallenbach et al., 2002). Ren (2002) believed that grain was stored in forage. Cultivated grassland is utilized efficiently and rationally by grazing livestock rather than through the harvest of seeds and hay. The production of white clover (*Trifolium repens*) through continuous grazing was significantly lower than that of rotational grazing at the same grazing intensity (Evans and Williams, 2010). Under mixed sowing, there were both competition and promotion effects among species. Due to the different growth characteristics of gramineae and legumes, they grow at different heights, thus improving light interception and conversion efficiency (Eric et al., 2003). For example, a study in the mountainous areas of Western Europe showed that mixed sowing of *Lolium perenne* + *T. repens*, *Trifolium pratense*, and *Plantago asiatica* increased yield ratios by 36, 37, and 35%, respectively, compared with monoculture (Tozer et al., 2016). In grassland cultivation, different use modes, intensities, and periods will affect the yield and quality of forage, thereby affecting the growth and development of herbivorous livestock and the production of livestock products. Selective feeding of animals (grazing preference) can change the competitiveness of forage species. On the one hand, animal feeding directly affects the growth and reproduction of forages, and the biomass of forage plants with strong grazing tolerance (supercompensation ability) increases after grazing. On the contrary, the growth and reproduction of forage plants with poor grazing tolerance are inhibited, resulting in the gradual disappearance of these plants from the community. On the other hand, livestock selectively feeds on palatable, high-quality grass (Wang et al., 2013). In addition, by using compensatory and balanced growth characteristics of plants, mowing cultivated grassland can promote forage growth, affect forage yield, and change the distribution of forage nutritional quality and deposition (Iraj and Sharrow, 1990).

The arid and semi-arid region in northwestern China that serves as an important agro-pastoral ecotone, this region has historically struggled with feed supply and ecological barriers. To develop potential solutions to this predicament, experiments were carried out in the typical areas in a rainfed agroecosystem, which monitored the yield, nutrient quality and soil nutrients, under two sowing modes and different cutting modes. Here, the following questions were addressed: (1) how does simulated grazing and harvesting hay treatments influence annul forage yield and herbage quality? and (2) How did two sowing method and different mowing patterns affect soil nutrient dynamics? and (3) establish and verify the model of forage nutrient quality from forage yield. The aim of this study is to identify methods to better utilize cultivated grassland in the Loess plateau.

## Materials and Methods

### Study Site

**Figure 1:**
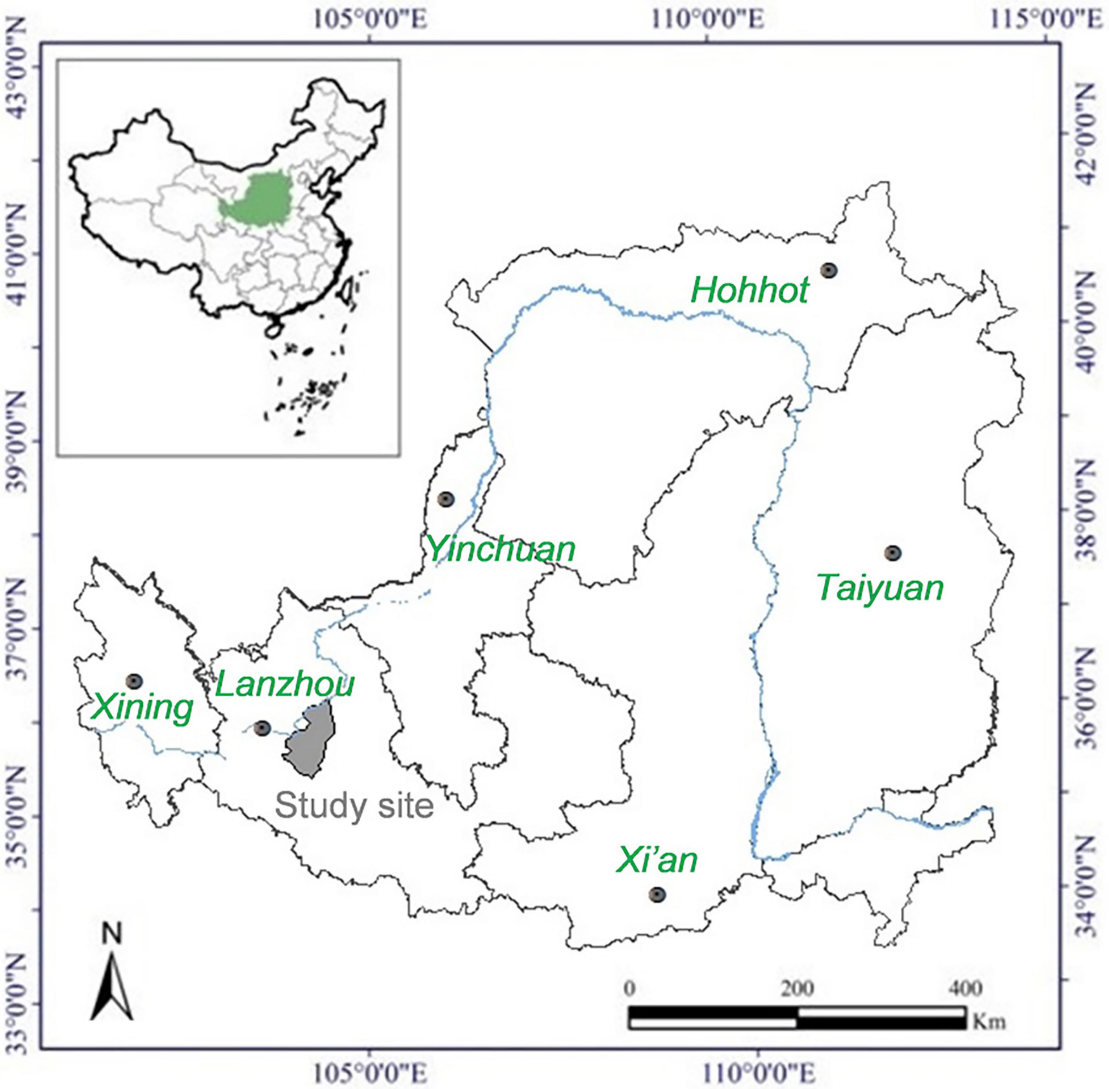
Location of study site on the Loess Plateau.

**Figure 2:**
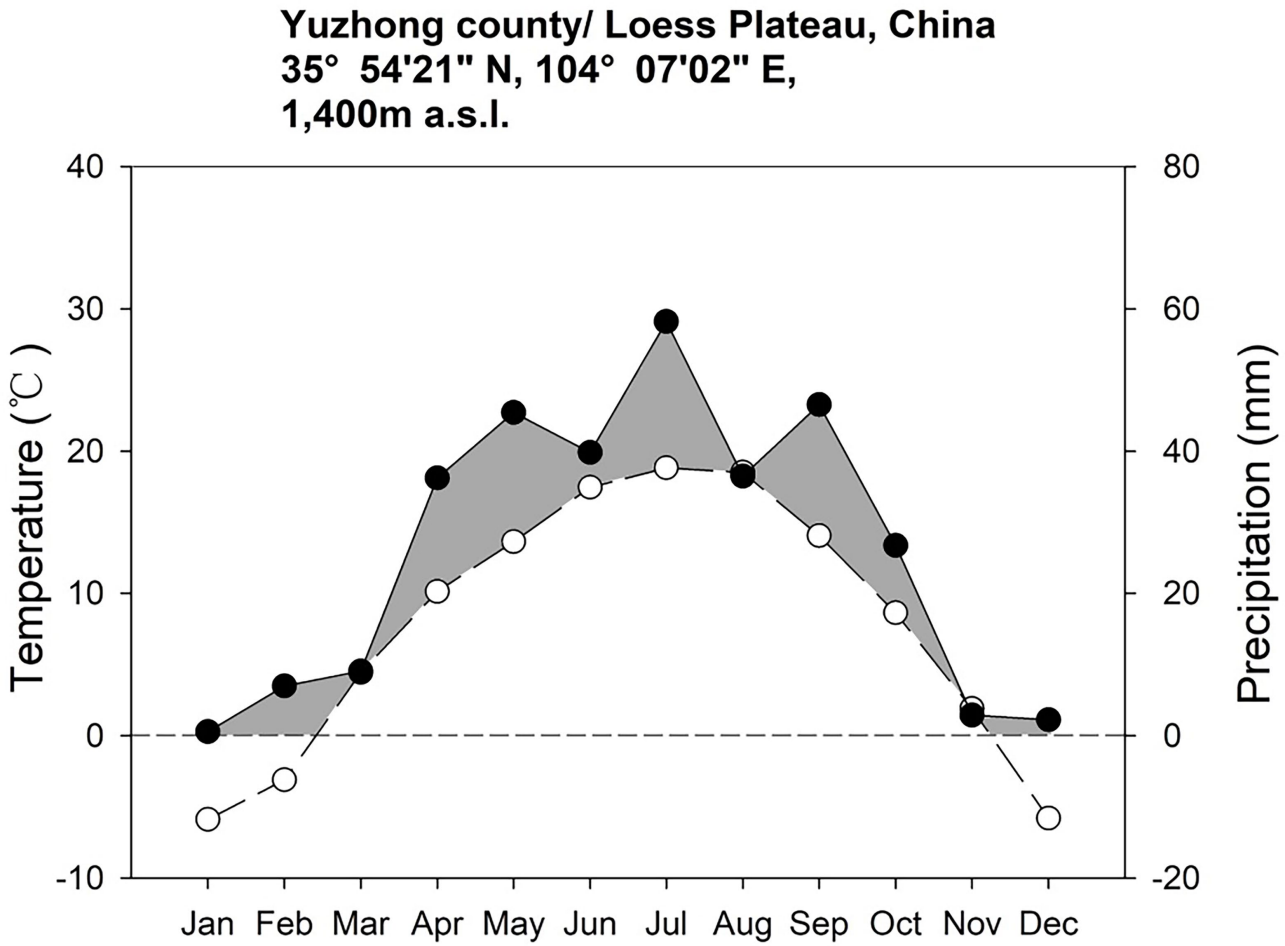
Mean month air temperature (mm, bars) and precipitation rates (°C, lines) measured at Yuzhong county research station (Lanzhou city, Gansu province, China) from 2013 to 2016. This figure uses the ratio of 10°C vs. 20 mm as in the standard Walter-Lieth climate diagrams.

The study site (latitude 35°54′21″N, longitude 104°05′02″E, elevation about 1,407 m) is located on the Loess Plateau in the Yuzhong County, Lanzhou City, Gansu Province, China (Figure 1). The growing season is from April to October and the out of season is from November to December. Mean annual temperature is 6.7°C, mean daily temperature is −5.5°C in January and 18.2°C in July. Mean annual precipitation is 388 mm, falling mostly in July and August (Figure 2). There are on average 130 frost days per year. Annual cloud-free solar radiation is about 2,600 h. The soil of the study site is Cultivated loessial soils (World Reference Base for Soil Resources; Food and Agriculture Organization of the United Nations (FAO), 2020).

### Experimental Design

**Figure 3:**
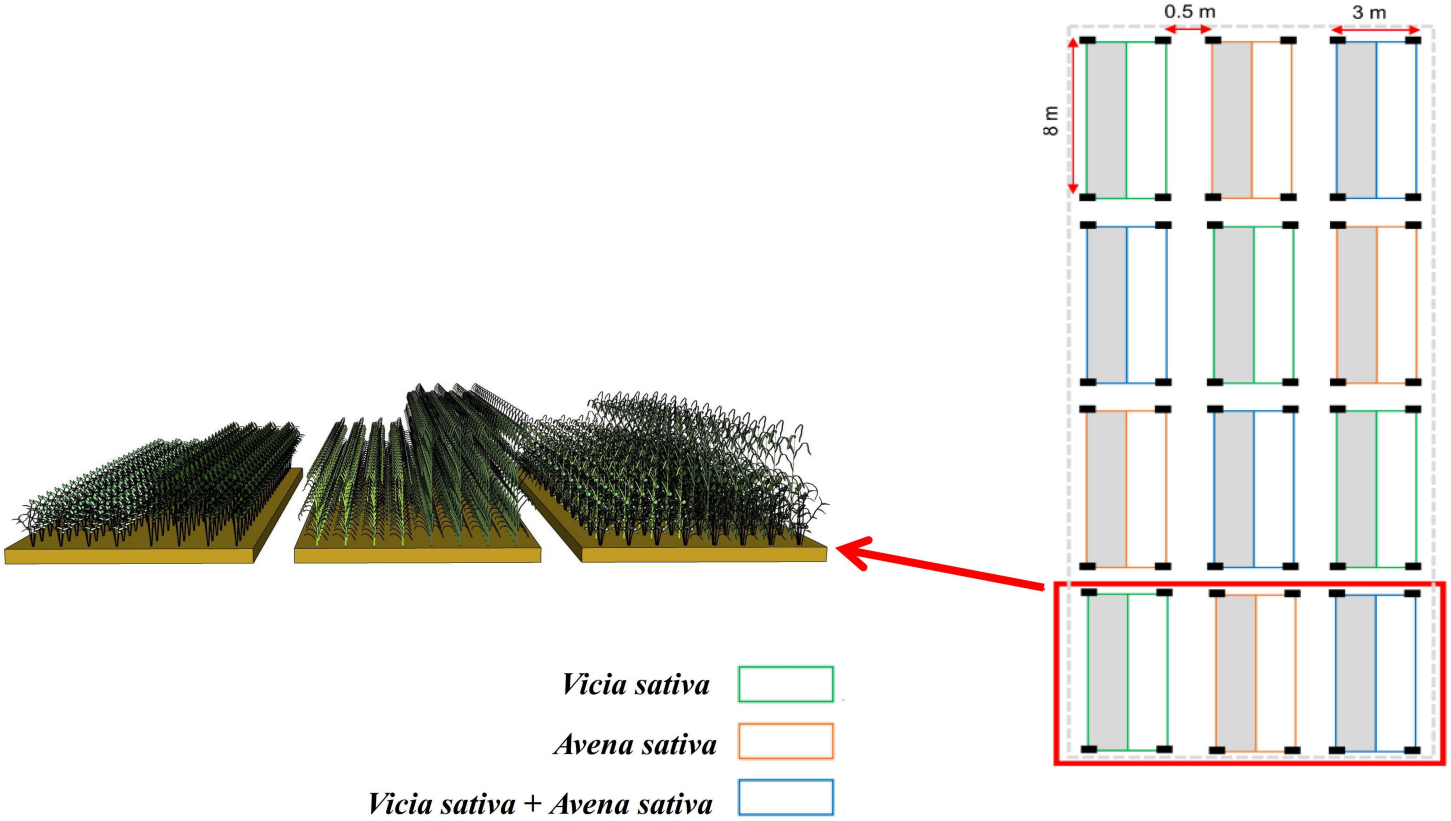
Layout of field experimental design. Left half in gray represents simulated grazing treatment; right half in white represents harvesting hay treatment.

The cultivated forage varieties were *Vicia sativa* (Leguminosae) and *Avena sativa* (Gramineae). The plot trial was established in 2014 and conducted in the same year. Before sowing, the fields were tilled, weeded, irrigated, and fertilized. Tillage depth was about 30–40 cm, fertilizer rate was 150 kg/hm^2^ (diamine) and 300 kg/hm^2^ (urea). In 2014, farmland with flat terrain was selected as the experimental plot, and the complete random block design was adopted. Three varieties [combinations (*Vicia sativa*, *Avena sativa*, and *Vicia sativa* + *Avena sativa*)] were tested, with four replicates, resulting in a total of 12 plots with 3 × 8 m each (Figure 3), there is 50 cm line protection, there are 1 m interval betwween the area. The experiment simulated livestock grazing by regular mowing, and the forage was divided into two use methods: simulated grazing (SG) and traditional hay harvesting (H). Harvesting was performed in October, leaving a stubble height of 10 cm. *Vicia sativa* and *Avena sativa* seeds were purchased from Beijing Zhengdao Seed Industry Co., Ltd.

### Plant Sampling

Sampling time should be consistent with each cutting time. Sampling should be done every 40 days in SG mode, starting from May, 3–5 times per year depending on the actual growth conditions. Forage yield: The fresh weight and dry weight of forage were measured by cutting method with 10 cm stubble and 1 m^2^ sample frame. The forage was divided into two parts. One part was dehydrated at 105°C, and dried at 85°C for 72 h to measure the dry weight (kg/m^2^). The other part was dried at 65°C for 48 h, and then crushed and sieved for routine nutrient analysis of forage. Crude protein (CP) content was determined by Kjeldahl method; crude fat (EE) was measured by ANKOMAXT15i automatic fat analyzer. The contents of neutral detergent fiber (NDF) and acid detergent fiber (ADF) were determined by filter bag and technology, and the instrument was ANKOM A200i semi-automatic fiber analyzer. Crude ash (ASH) content was determined by high temperature burning method (TM-O91OP muffle furnace).

### Soil Sampling

The soil is sampled at the same time as the aboveground portion. Soil samples were collected from 0 to 10 cm, 10–20 cm and 20–30 cm soil layers according to the distribution of grass roots in five sites randomly selected in each plot. The soil samples were filtered through 2 mm soil sieve and air dried for soil nutrient determination. Indicators of soil organic carbon, total nitrogen, NO_3_^−^-N and NH_4_^+^-N refer to ‘Soil Agrochemical Analysis’ (Bao, 2000).

### Data Collection

Microsoft Excel 2010 software was used to process the relevant data. SPSS 20.0 statistical analysis software was used to analyze the correlation and LSD test of the data (Zhang et al., 2019). Origin 2018 was used to plot soil nutrient data. RDA and boxplots were drawn using R version 3.6.1 [vegan (Halvorsen et al., 1999) and stats (David, 1972) packages]. Technique for Order Preference by Similarity to an Ideal Solution (TOPSIS) was first proposed by Wang and Yoon in 1981 (Wang et al., 2021). TOPSIS is a method to sort the limited evaluation objects according to their proximity to the ideal target, which is to evaluate the relative advantages and disadvantages of the existing objects. TOPSIS method is a kind of ordering method that approximates to ideal solution, which only requires that each utility function has monotonically increasing (or decreasing) property. TOPSIS method is a common and effective method in multi-objective decision analysis (Yu et al., 2022).

## Results

### Forage Yield

**Figure 4:**
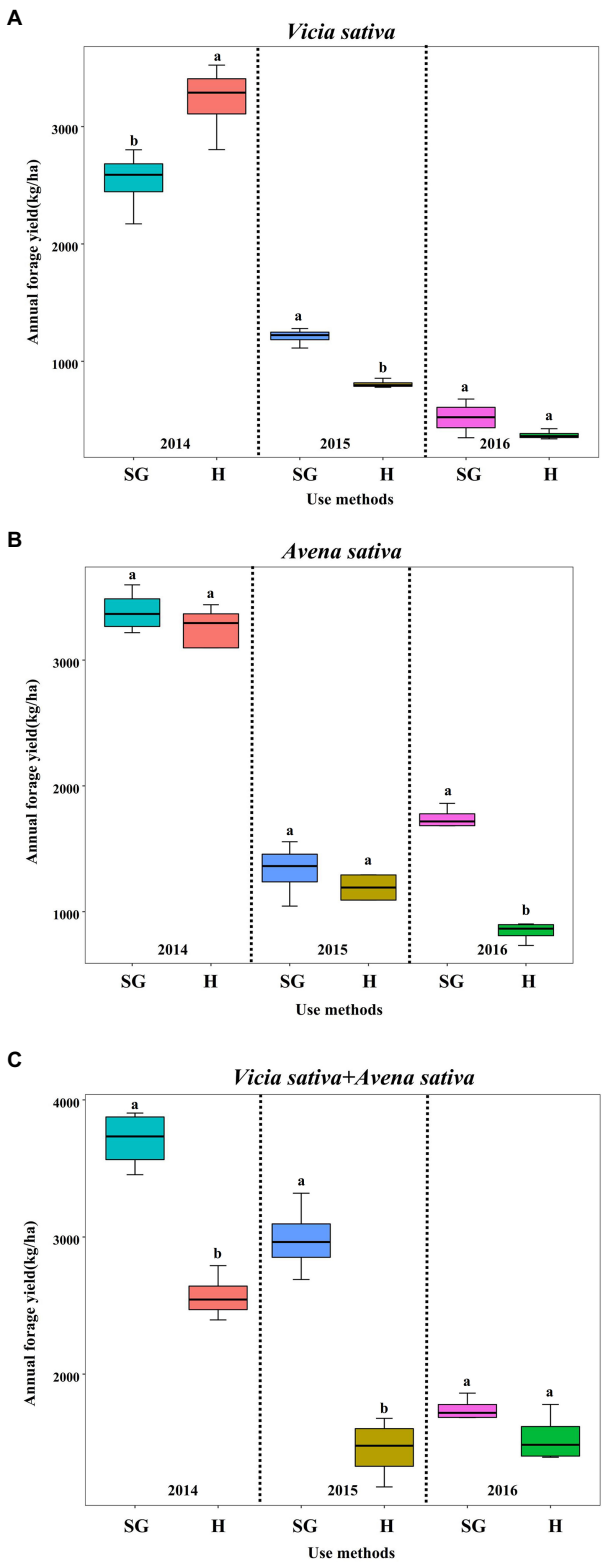
Response of annual forage yield (kg/ha) of three forage crops to different use methods (SG: simulated grazing and H: harvesting hay) in 2014–2016. **(A)** Vicia sativa, **(B)** Avena sativa, **(C)** Vicia sativa +Avena sativa. Different small letters indicate significant differences (*p* < 0.05), and other cases with the same letter or no letter showed no significant difference (*p* > 0.05).

As shown in Figure 4, in 2014, under simulated grazing, the grass yields of *Avena sativa* (Figure 4B) and mixed *Vicia sativa + Avena sativa* (Figure 4C) were higher than those of harvested hay, and the yield of mixed *Vicia sativa + Avena sativa* was highest (*p* < 0.05), i.e., 1,138.00 kg higher per hectare. In 2015, the yield of each forage variety (combination) was higher than that of harvested hay. The yield of common *Vicia sativa* (Figure 4A) and mixed *Vicia sativa* + *Avena sativa* (Figure 4C) were significantly higher (*p* < 0.05), namely 402.23 and 1,531.93 kg per hectare, respectively. In 2016, the yield of each forage variety (combination) was higher than that of harvested hay, and the yield of *Avena sativa* (Figure 4B) was highest (*p* < 0.05). At the same time, the yield of the combination was higher than that of single forage varieties, indicating an advantage of mixed sowing.

**Figure 5:**
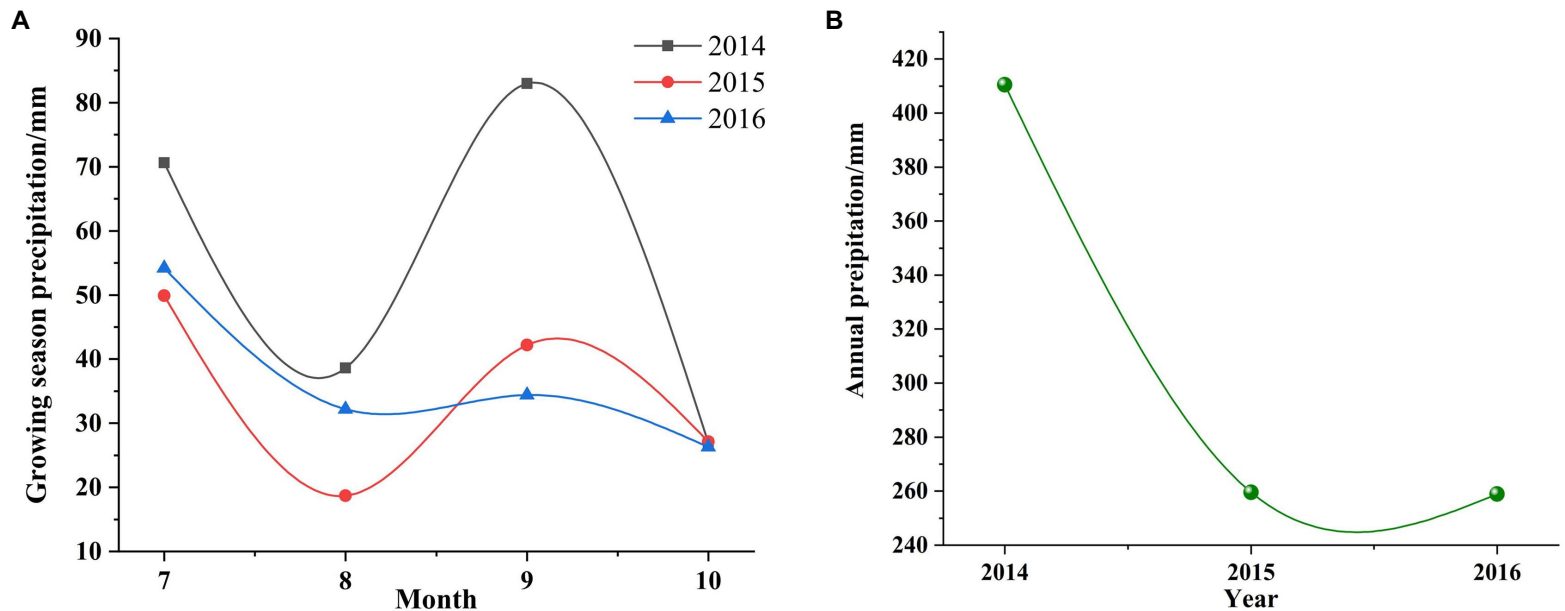
Precipitation during the growing season **(A)** and Annual Precipitation **(B)**, 2014–2016.

The analysis of yield results of forage varieties (combinations) between different years showed that the yields of *Vicia sativa* and *Avena sativa* in 2014 were significantly higher than those in 2015 and 2016, and the yields of *Vicia sativa + Avena sativa* under two different cutting modes were also higher than those in 2015 and 2016, respectively. The detection results of Precipitation (Figure 5) from 2014 to 2016 showed that the Precipitation in 2014 (Figure 5B) reached 410.4 mm, which was significantly higher than that in 2015 and 2016. Precipitation in growing season (Figure 5A) was also higher than that in 2015 and 2016, which may be the main factor leading to yield differences in different years.

### Herbage Quality

From 2014 to 2016 (Table 1), analysis of forage quality results showed that. In 2016, CP and EE contents of all herbage varieties (combinations) were higher than those in 2014 and 2015. Meanwhile, CP and EE yields of three herbage varieties (combinations) in SG mode were higher than those in H mode. Among them, *Vicia sativa* showing the highest value (*p* < 0.05). The ASH content was lower than that of the harvested hay, and *Avena sativa* and the *Vicia sativa* + *Avena sativa* combination showed the highest values (*p* < 0.05). In 2014, the CP content of each variety and combination under simulated grazing was higher than that under hay harvesting, with the highest values found for *Avena sativa* (*p* < 0.05), 35.65% higher than that under hay harvesting. The CP content of *Vicia sativa* + *Avena sativa* was significantly higher than that of *Avena sativa* (*p* < 0.05), whereas the ASH content of different combinations under hay harvesting was significantly higher than that under simulated grazing (*p* < 0.05); there were no significant differences in the other indicators (EE, NDF, and ADF). In 2015, the CP contents of *Vicia sativa*, *Avena sativa*, and mixed *Vicia sativa* + *Avena sativa* combinations under simulated grazing were significantly higher than that of harvested hay, with increases by13.50, 51.99, and 40.40%, respectively. The EE content of *Vicia sativa* under simulated grazing was significantly higher than that of harvested hay (*p* < 0.05). The ASH contents of *Vicia sativa*, *Avena sativa*, and mixed *Vicia sativa* + *Avena sativa* were significantly lower than that of harvested hay under simulated grazing (*p* < 0.05).

**Table 1:** Response of different forage crop cultivation methods to forage varieties under different use patterns in 2014–2016 (*Vicia sativa*, *Avena sativa* or *Vicia sativa* + *Avena sativa*).

Nutritive contents (%)	Year	*Vicia sativa*		*Avena sativa*		*Vicia sativa + Avena sativa*	
		SG	H	SG	H	SG	H
CP	2014	15.99 ± 0.79a	15.07 ± 0.95a	7.8 ± 0.43d	5.75 ± 0.43e	9.39 ± 0.52bc	8.36 ± 1.03 cd
	2015	12.33 ± 0.84a	10.86 ± 0.83b	9.09 ± 0.41c	6.02 ± 0.36d	9.93 ± 0.69bc	7.83 ± 1.12d
	2016	20.86 ± 1.44a	18.32 ± 0.96b	12.76 ± 0.55d	11.57 ± 0.53d	15.16 ± 0.54c	14.99 ± 1.52c
EE	2014	0.99 ± 0.48c	1.09 ± 0.55c	2.11 ± 0.70b	2.34 ± 0.86ab	2.92 ± 0.07a	2.51 ± 0.50ab
	2015	2.41 ± 0.04ab	1.17 ± 0.09c	2.63 ± 0.34ab	2.14 ± 0.46ab	3.02 ± 0.43a	2.54 ± 0.49ab
	2016	3.38 ± 0.70b	2.37 ± 0.14c	4.69 ± 0.71a	4.09 ± 0.19a	4.23 ± 0.32a	4.37 ± 0.24a
ASH	2014	5.59 ± 0.51c	7.72 ± 0.38ab	6.60 ± 0.84b	8.27 ± 0.68a	6.02 ± 0.94c	7.70 ± 0.33ab
	2015	7.59 ± 0.75b	11.79 ± 0.86a	6.61 ± 0.26c	11.26 ± 2.13a	7.45 ± 0.12b	12.35 ± 2.81a
	2016	8.22 ± 0.49b	9.65 ± 0.66ab	7.81 ± 1.39c	11.08 ± 0.57a	8.24 ± 0.47bc	10.51 ± 0.70a
NDF	2014	41.40 ± 4.15c	47.63 ± 2.86b	46.77 ± 1.27bc	52.46 ± 0.66a	50.51 ± 2.42a	50.27 ± 1.02a
	2015	54.57 ± 1.98a	46.63 ± 2.22b	46.69 ± 0.75b	51.20 ± 1.43ab	50.00 ± 2.19ab	46.52 ± 1.26b
	2016	35.16 ± 2.08c	34.77 ± 2.12c	34.69 ± 1.32c	39.91 ± 3.79b	43.14 ± 2.06a	40.87 ± 1.14a
ADF	2014	28.74 ± 0.06d	25.34 ± 1.86 cd	27.87 ± 3.72 cd	30.39 ± 0.87c	37.26 ± 1.55b	47.53 ± 0.81a
	2015	47.04 ± 1.71a	40.20 ± 1.91c	30.51 ± 0.49d	43.60 ± 2.08b	32.29 ± 1.55	42.53 ± 0.81bc
	2016	34.50 ± 1.21bc	38.71 ± 2.05a	30.18 ± 2.52c	31.48 ± 1.64c	35.35 ± 1.25b	41.45 ± 1.77a

*In the same row, no letters or the same letters indicate no significant difference (*p* > 0.05), the same as below. In the same line, different small letters indicate significant differences (*p* < 0.05), and other cases with the same letter or no letter showed no significant difference (*p* > 0.05), the results were expressed by “Mean ± SD.”*

### Relationships Between Forage Yield and Herbage Quality

**Figure 6:**
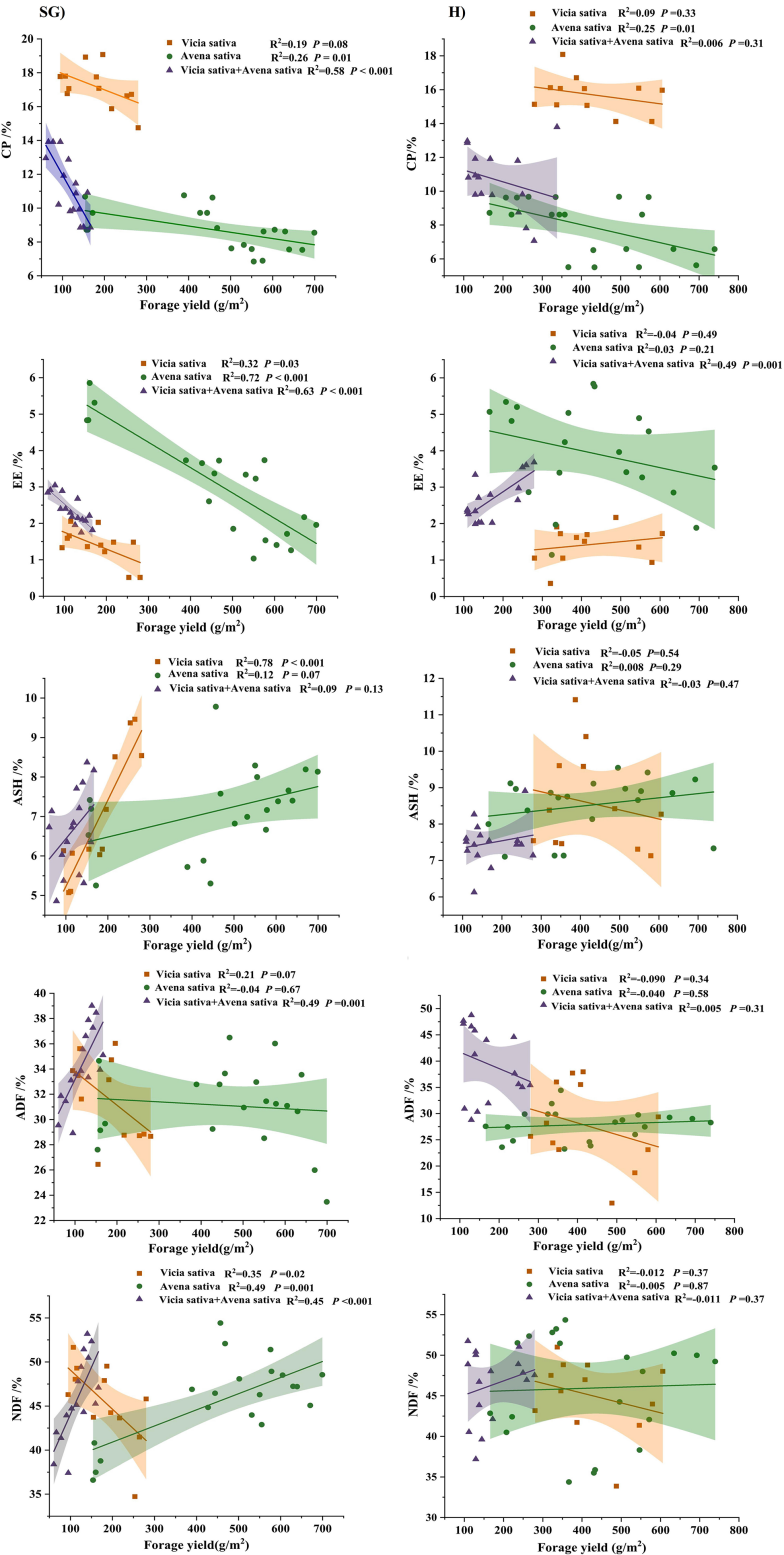
Relationships between forage yield and crude protein (CP), crude fat (EE), Crude ash (ASH), neutral detergent fiber (NDF) and acid detergent fiber (ADF) under simulated grazing and harvesting hay treatments. Lines denote the linear fit.

As shown in Figure 6, Regarding the CP content, under the different use modes, with increasing forage yield, the CP contents of legume and gramineous forage decreased, showing a linear negative correlation, and the difference in the decrease between *Avena sativa* and *Vicia sativa* + *Avena sativa* was significant (*p* < 0.05). In terms of the EE content, under simulated grazing, the value of the combination of the three species decreased significantly (*p* < 0.05), showing a linear negative correlation; under the hay harvest mode, the EE content of monoculture *Avena sativa* decreased with increasing yield, whereas that of *Vicia sativa* and the mixture (*Vicia sativa* + *Avena sativa*) increased with increasing yield. In terms of ASH content, there was a positive correlation between the forage yield of different species under simulated grazing condition, and the content of alfalfa and blue and white alfalfa was the highest (*p* < 0.05); under hay harvesting mode, except *Vicia sativa* showed a significant negative correlation, the others were positively correlated. In terms of the ADF content, under simulated grazing, monocultures of *Vicia sativa* and *Avena sativa* showed decreasing values at increasing forage yield, and with the lowest values for *Avena sativa*. Under hay harvesting, *Avena sativa* showed a downward trend, whereas *Vicia sativa* and the mixed sowing combinations showed an increasing trend. Regarding the NDF content, under simulated grazing, with the increase in forage yield, the values for *Avena sativa* and *Vicia sativa* + *Avena sativa* significantly increased (*p* < 0.05), whereas those for *Vicia sativa* decreased (*p* < 0.05).

### Redundancy Analysis Between Climatic Factors and Forage Yield and Nutritional Quality

**Figure 7:**
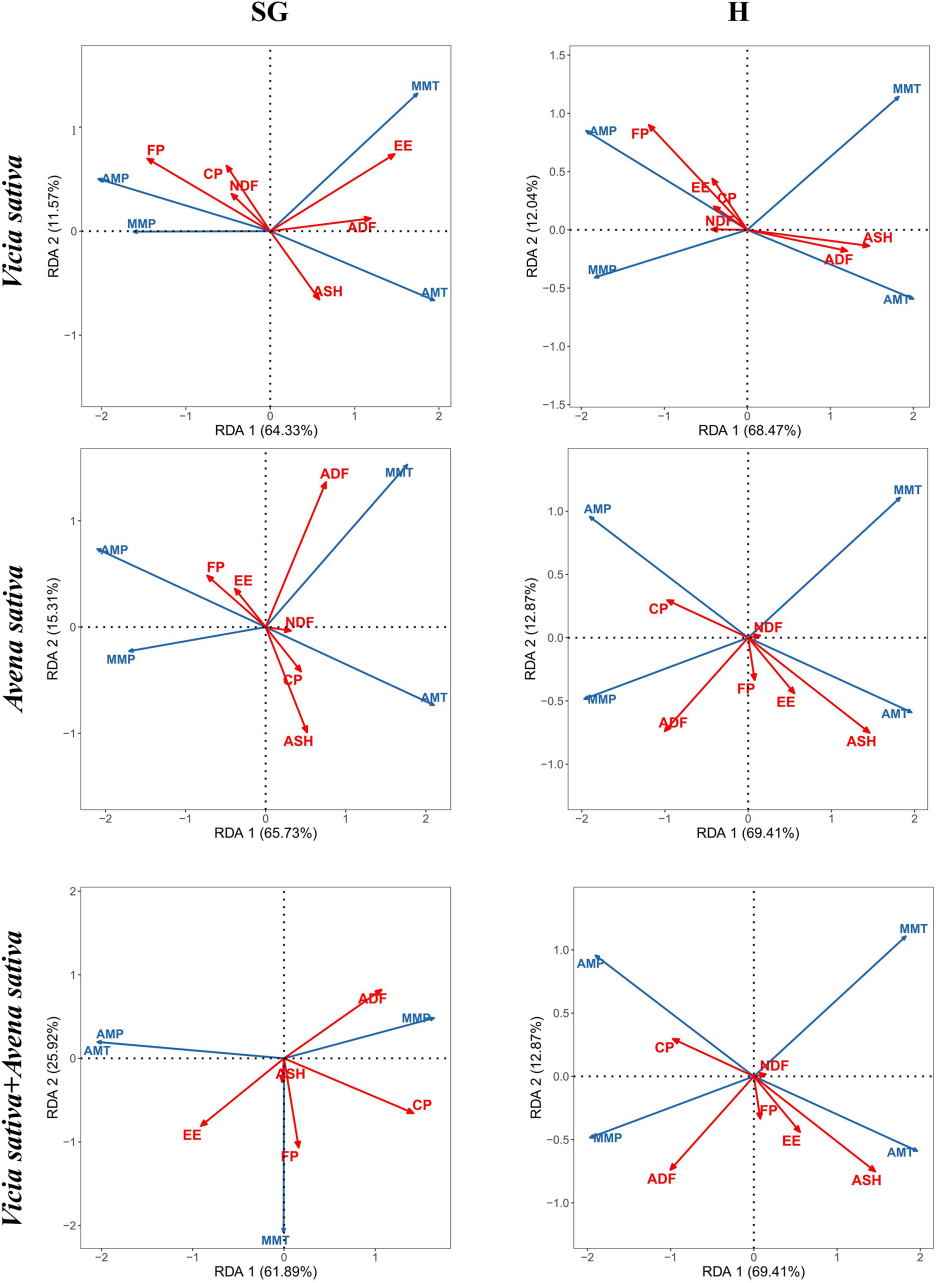
Biplot of the first two axes of the distance-based redundancy analysis for forage yield and herbage quality associated with various ecological characteristics under simulated grazing. FP, forage yield; AMT, annual mean temperature; AMP, annual mean precipitation; MMT, month mean temperature; MMP, month mean precipitation.

Redundancy analysis (RDA) can be used to determine the influence of environmental factors on samples and to verify the relationship between samples and environmental factors. Figure 7 shows the relationships among sample, forage yield (FP), and forage nutritional value. The total interpretation of each forage variety combination under the two use methods exceeded 60%. In general, there was a positive correlation between annual precipitation and crude protein and grass yield. Under simulated grazing, the contents of EE and ASH in *Vicia sativa* were positively correlated with AMT and MMT. In the mixed sowing combination, annual precipitation was negatively correlated with CP and FP and positively correlated with the average monthly precipitation in the growing season. Based on the results, MMP had a considerable impact on forage growth. Under hay harvesting, AMT was positively correlated with ADF and ASH of *Vicia sativa*. Similarly, the ASH content in *Avena sativa* and mixed sowing combinations was positively correlated with AMT.

### Dynamic Changes of Soil Nutrients in Different Forage Varieties (Combinations)

**Figure 8:**
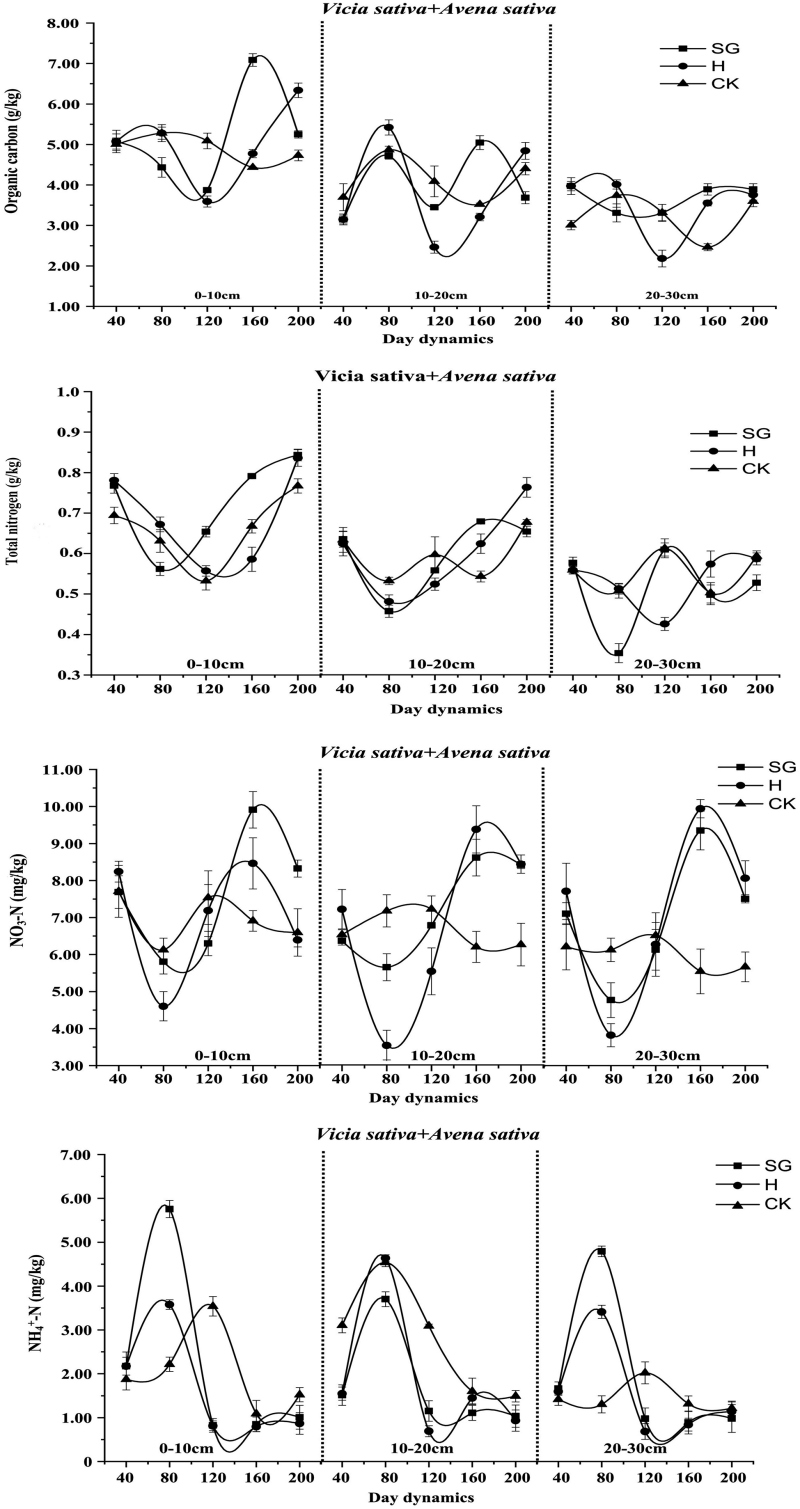
Dynamic changes of soil nutrients of *Vicia sativa*.

In *Vicia sativa* (Figure 8), with increasing soil depth, the levels of soil organic carbon and total nitrogen decreased gradually. The soil nutrient levels differed considerably between the two use methods, albeit with a similar change trend. The CK level changed only slightly over time, and its influence was highest in the 0–20 cm soil layer. Under simulated grazing, except NH_4_^+^-N, all nutrient contents decreased first and then increased. Also, under simulated grazing, although more soil nutrients were consumed in the early stage, in the later stage, there was a certain return effect, which was more obvious in the 0–10-cm layer.

**Figure 9:**
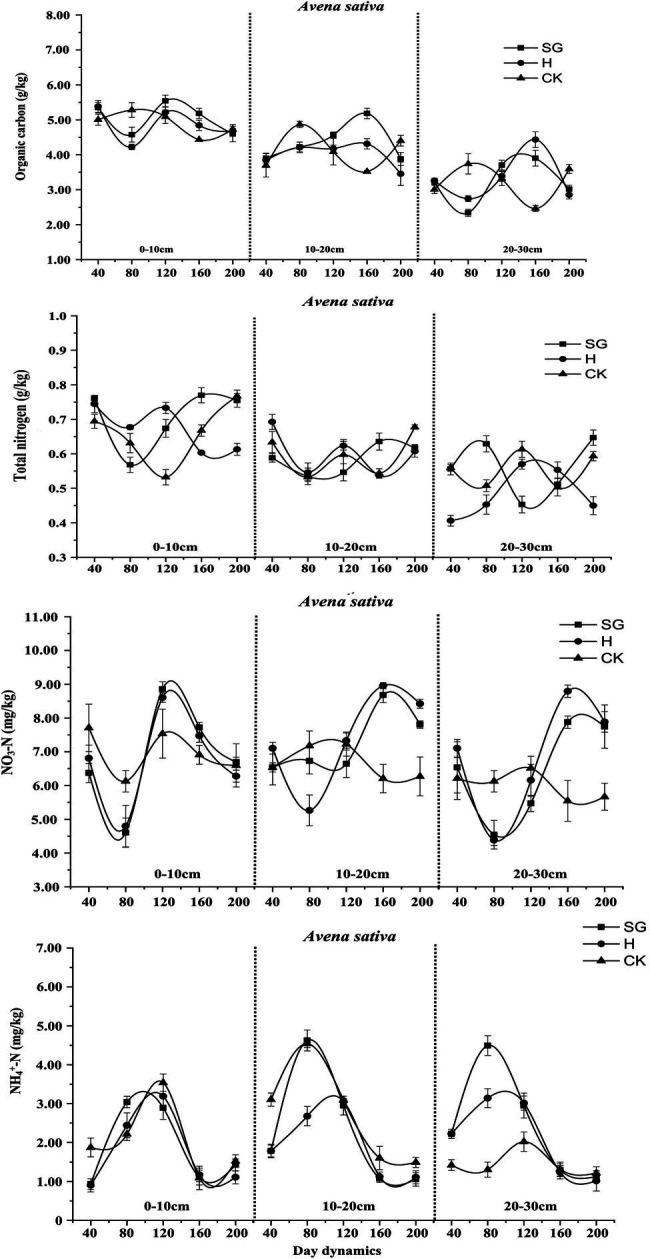
Dynamic changes of soil nutrients of *Avena sativa*.

For *Avena sativa* (Figure 9), similar to *Vicia sativa*, soil organic carbon and total nitrogen content decreased with increasing soil depth, and the soil nutrient content remained consistent after the last cutting before planting. The soil nutrient content in CK changed slightly over time and tended to be stable. The contents of soil organic carbon, total nitrogen, and NO_3_^−^-N decreased first, then increased, and then decreased, which was most obvious for the hay harvesting mode, most likely because of the late reproductive growth of *Avena sativa* under this mode. The change trend of the NH_4_^+^-N content in monocultures of *Vicia sativa* and *Avena sativa* was similar.

**Figure 10:**
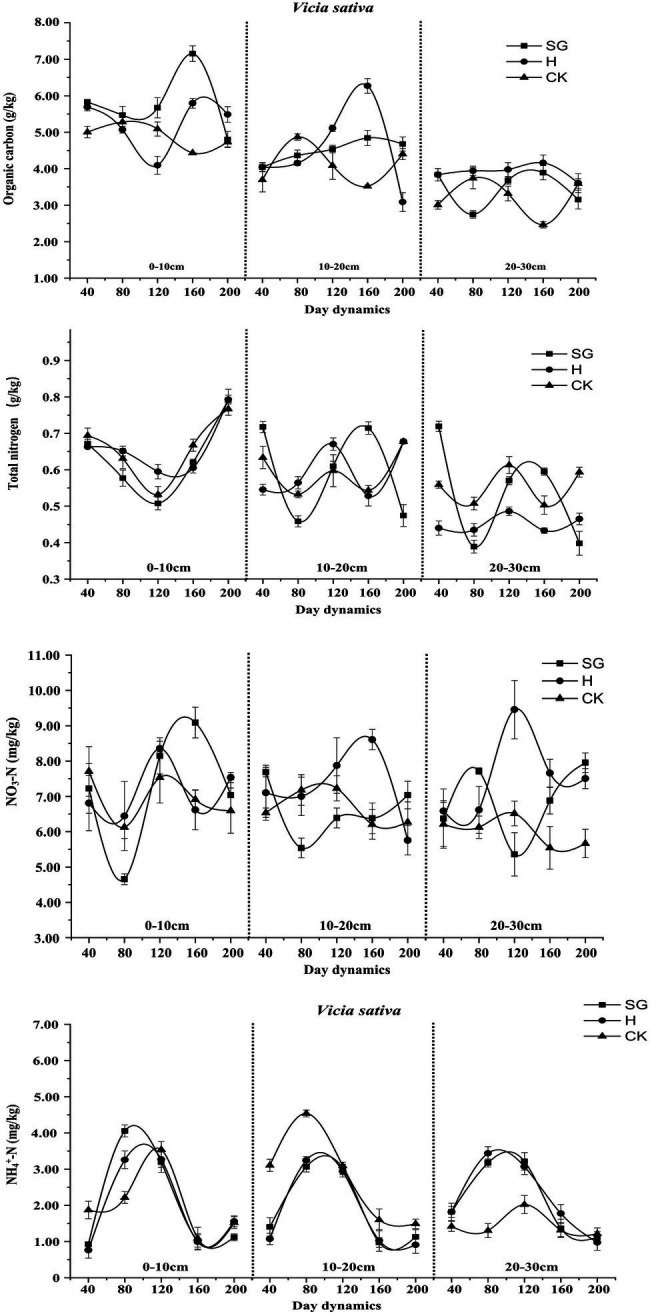
Dynamic changes of soil nutrients of *Vicia sativa + Avena sativa*.

For *Vicia sativa* + *Avena sativa* (Figure 10), the variation trend of the soil nutrient contents over time was similar to those of *Vicia sativa* and *Avena sativa*. Under mixed sowing, the changes in the NH_4_^+^-N content in each soil layer also tended to be consistent, but the consumption of NH_4_^+^-N was high during the growth period.

### Comprehensive Evaluation Value of TOPSIS

**Figure 11:**
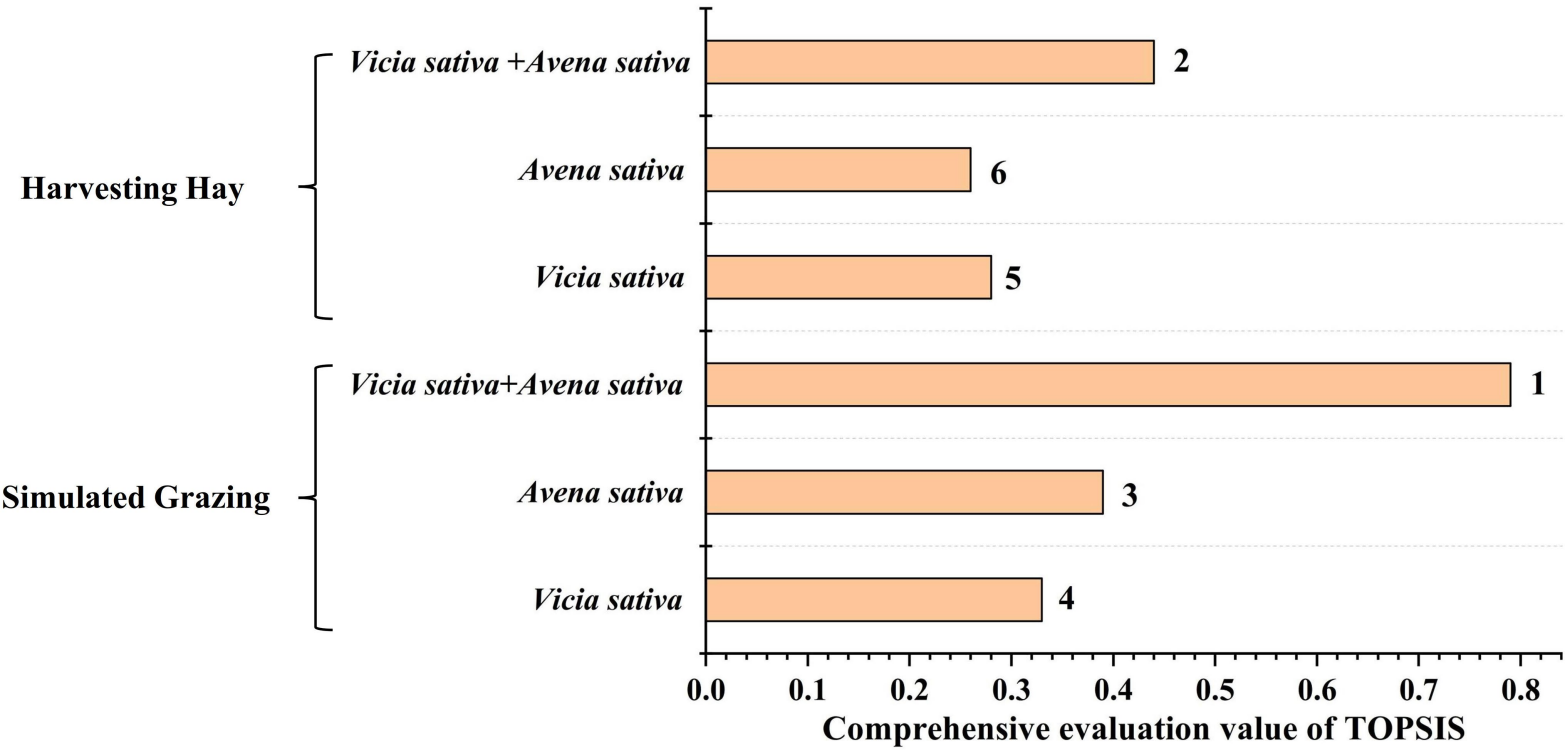
Comprehensive evaluation of cultivated grassland with different sowing varieties (combinations) under simulated grazing (SG) and hay harvesting (H).

The comprehensive evaluation values of TOPSIS (Figure 11) were determined under simulated grazing and hay harvesting. The TOPSIS comprehensive evaluation values of monoculture *vicia sativa*, *Avena sativa* and *Vicia sativa + Avena sativa* were 0.33, 0.39, and 0.79, respectively under simulated grazing, whereas the values of monoculture *vicia sativa*, *Avena sativa*, and *Vicia sativa + Avena sativa* were 0.28, 0.25, and 0.44, respectively. According to the comprehensive evaluation value calculated by TOPSIS, mixed sowing provided better results than monoculture, and simulated grazing was better than hay harvesting.

## Discussion

### Effects of Different Use Modes on Forage Yield and the Nutritional Quality of Different Forage Varieties (Combinations)

The yield of herbage is an important indicator to measure grassland resources, which determines the amount of food provided by grassland for livestock and has a great influence on the carrying capacity of grassland (Kawamura et al., 2008). In this study, the growth and nutritional value characteristics of pasture under two use methods (simulated grazing and harvesting hay) and different planting methods (single sowing and mixed sowing) were quantitatively analyzed. We found that, compared with hay harvesting, simulated grazing can improve the yield of high-quality forage and has a greater advantage for the forage value of various combinations. Volenec et al. (1987) showed that plant height, tiller number and plant density of forage had extremely significant effects on forage yield (*p* < 0.01), among which tiller number had the greatest effect. Yang et al. (2015) simulated rotational grazing with multiple mowings showed that the tiller number, plant height and grass yield of four ryegrass varieties increased with the increase of mowing times, and multiple mowings significantly increased the yield of all ryegrass varieties (*p* < 0.05). This may be because multiple cutting can relieve the top advantage of forage, and forage has compensatory growth characteristics, thereby increasing the yield of forage. Lei et al. (2005) showed that multiple cutting was beneficial to the increase of yield in oat growth period. The reason may be that oats can relieve the apical dominance and stimulate the growth of stems and leaves under multiple cutting conditions, so as to increase yield. This study was consistent with the conclusion that the total hay yield under multiple cutting was significantly higher than that under conventional cutting.

Different planting patterns and use patterns affect forage yield and nutritional quality in grassland cultivation (Yang et al., 2015). The nutritional quality of forage can not only directly affect the growth, reproduction, forage-herbivore interaction, and foraging behavior of livestock and wild herbivores by affecting the difficulty in obtaining nutrients, but can also indirectly affect the yield, quality, and economic benefits of livestock products (Waterman et al., 2007; Cui et al., 2016). From the point of view of nutritional value, CP is an essential nutrient for livestock. Its content not only affects the economic benefits of forage but also directly affects the milk yield and milk protein yield of livestock (Yang et al., 2017). From the cutting period of wheat crops, with the extension of growth period, the crude protein content decreased and the crude fiber content increased (Sun, 2003). The simulated grazing method can effectively increase the CP content and has the advantage of mixed sowing. The forage quality of each variety decreased gradually with the maturity of the plant under the dry-harvesting mode, most likely because with plant maturity, the forage litter increased and the ratio of stem to leaf increased, resulting in a decreased nutritional quality. Simulated grazing effectively promoted the tillering of forage, reduced the loss of forage litter, and thus improved the nutritional quality of forage. In addition, young plant tissue accounted for a large proportion after short stubble, with a high nutritional value (Inyang et al., 2010). Yang et al. (2015) found that compared with conventional hay harvest, multiple cutting significantly reduced the crude fiber content of ryegrass (*p* < 0.05), and significantly increased the crude protein, crude fat and crude ash content (*p* < 0.05). Moreover, multiple cutting treatments increased the grass yield, crude protein and crude fat yield of the three high-sugar ryegrasses, and the results of this study were similar to those above.

### Relationship Between Forage Yield and Nutritional Quality

Forage yield represents the relationship between forage and the environment (Lauer et al., 2001), whereas forage quality represents the use efficiency, impacts forage digestion and absorption, as well as energy intake and nutrient acquisition, and affects the yield and quality of livestock products (Richman et al., 2014). Generally, there is a correlation between forage quality and yield. White and Wight (1984) reported that the crude protein content decreased by 0.80–1.25%, and the digestible dry matter content decreased with each 1,000 kg/ha increase in grass yield. In alfalfa, with continuous cultivation, the biomass of leaves and stems increases, accounting for 60 and 40% of the total yield, respectively (Pavlů et al., 2006). In the present study, there was also a certain correlation between forage nutritional quality and forage yield. Grass yield was negatively correlated with CP content and EE and positively correlated with ASH content, that is, the increase in grass yield led to the decrease in forage nutritional value. In other words, with increasing grass yield, the nutritional value decreases. The reason may be that with the maturity of forage grass, the digestibility of the stem decreased and the fiber content increased. When the forage grass was mature, the relative proportions of leaf and stem decreased (Tahmasebi et al., 2019). The distribution of various elements during plant growth may affect the relationship between forage quality and forage yield. From the perspective of ecological stoichiometry, higher biomass in plant communities means “dilution” of nutrient elements such as N and P, resulting in the decrease of nutrient elements with the increase of biomass (He et al., 2006; Reich et al., 2006). Specifically, it is because with the accumulation of dry matter in plants, the proportion of non-mechanical tissues that are physiologically active gradually decreases with the continuous growth of mechanical tissues (Niklas et al., 2005). Due to the low content of N in mechanical tissue, but the content of cellulose, lignin and other crude fiber components is more, resulting in the increase of grass yield, crude protein decreased and crude fiber increased.

### Relationship Among Climatic Factors, Forage Yield, and Nutritional Quality

On the one hand, the nutrient content of forage is affected by the physiological characteristics, on the other hand, it is restricted by the environmental conditions in the growing place, which is the result of the interaction of species phylogeny and environmental factors (Aerts and Chapin, 1999). Therefore, forage quality is closely related to environmental factors.

In the rain-fed agricultural system, rainfall plays a decisive role, and the amount of water determines the survival, growth, and reproduction of plants. Studies have shown that changes in rainfall have significant effects on plants. Drought affects plant phenology, and delays flowering and even vegetative growth (Zhang et al., 2010). For some wild perennial gramineous plants, in the case of high-rainfall growing seasons, reproductive organ reconstruction has frequently been observed, and the relative reduction of nutrients used for reproduction reduces the proportion of the reproductive distribution. Drought also affects the aboveground biomass of plants (Sman et al., 1993). For example, the decrease in precipitation increases the mortality of *Poa crymophila* in spring, significantly increases the content of carbon (C) in inflorescence, and significantly decreases the levels of C in the root. Therefore, in drought years, *P. crymophila* plants are smaller, allocate more biomass to the aboveground structures and more C to the inflorescence. However, the levels of N did not change (Zhou et al., 2010). In this study, nutritional quality was positively correlated with rainfall and forage yield and negatively correlated with temperature. Previous studies have shown that elevated temperature reduces leaf/stem ratio and promotes the production of structural carbohydrates, resulting in increased crude fiber and reduced herbage digestibility (VcLzquez-de-Aldana et al., 2008). There was no significant correlation between nutritional quality and other environmental conditions.

### Dynamic Analysis of Soil Nutrients Under Different Use Patterns

In grassland ecosystem, soil is the most important substrate for plant growth and development, the reservoir of many nutrients and nutrient elements, and the site for microbial decomposition of plant and animal residues and material circulation. As a substrate for plant growth and development and an important environmental factor, soil physical and chemical properties have a significant impact on plant community dynamics (Janssens et al., 1998). Soil organic matter is an important index to measure the physical and chemical properties of soil, which has an important indication of soil quality change. The change of soil organic matter accumulation and decomposition rate will affect the carbon and nitrogen cycle in the ecosystem, and further affect the cycle and content of other nutrient elements in the system (Vinton and Burke, 1995). In this study, the contents of soil organic carbon, total nitrogen, NO3--N, and NH4 + -N in different forage varieties (combinations) under different use modes were significantly different; they first decreased, then increased, and then decreased again. The reason for this trend may be that a large amount of soil nutrients is consumed in the early growth of plants, resulting in a decrease in nutrient content. With the advent of the rainy season, soil nutrients are restored. In the later stage of plant growth, with the decrease of rainfall and continuous cutting, plant growth is promoted, and nutrient content in the soil is consumed again. Therefore, under simulated grazing, it is possible to supply a certain amount of soil nitrogen fertilizer in the early growth phase to accelerate growth. In this study, under simulated grazing, forage grass had a certain return effect on soil nutrients at the late growth stage; however, this effect on the shallow surface (0–10 cm) was more obvious under hay harvesting. This trend may be due to the reduction of litter and root exudates and the weakening of microbial activity caused by human disturbance (Zhang et al., 2016), resulting in lower soil nutrient levels. Li et al. (2002) carried out mowing treatment on *Medicago sativa*, *Sorghum sudanense*, and *Avena sativa* and showed that the soil total nitrogen content was lower than that of the hay harvest treatment, which was consistent with the results of this study on monoculture *Avena sativa* in the 0–20-cm soil layer.

## Conclusion

Results from the present study showed that among the two different utilization modes, compared with H mode, the forage yields of *Avena sativa* and *Vicia sativa + Avena sativa* increased by 216.78–903.5 kg/ha and 209.63–1,531.72 kg/ha, respectively, in SG mode from 2014 to 2016. The CP content of *vicia sativa*, *Avena sativa*, and *Vicia sativa + Avena sativa* increased 9.32–26.28%, 5.75–12.17%, 1.12–21.15%, respectively. The EE content increased 9.83–18.93%, 29.88–51.45%, 14.04–15.89%, respectively. The ASH content decreased 27.59–35.62%, 14.50–41.30%, 21.82–39.68%, respectively. The two methods had no significant effects on soil nutrient content, maintaining the relative stability of soil nutrient content. The comprehensive TOPSIS analysis showed that the effect of simulated grazing was better than that of hay harvest under the two utilization modes, and the effect of mixed sowing was better than that of single cultivation. Moreover, the RDA analysis showed that precipitation, especially in the growing season, was the key factor affecting the production performance of rain-fed agricultural cultivated grassland in the Loess Plateau.

## Data Availability Statement

The original contributions presented in the study are included in the article/supplementary material, further inquiries can be directed to the corresponding author.

## Author Contributions

FH designed and supervised the project. TL, LP, HW, and YC conducted the field work and collected the data. TL, YZ and FH wrote the manuscript with critical input from all the authors. All authors contributed to the article and approved the submitted version.

## Funding

The Program of National Science and Technology Assistance, Grant/Award Number: KY202002011; The Program for Innovative Research Team of Ministry of Education, Grant/ Award Number: IRT17R50; ‘Lanzhou City’s Scientific Research Funding Subsidy to Lanzhou University’. Key RESEARCH and Development Program of Ningxia Hui Autonomous Region (2020BBF02013).

## Conflict of Interest

The authors declare that the research was conducted in the absence of any commercial or financial relationships that could be construed as a potential conflict of interest.

## Publisher’s Note

All claims expressed in this article are solely those of the authors and do not necessarily represent those of their affiliated organizations, or those of the publisher, the editors and the reviewers. Any product that may be evaluated in this article, or claim that may be made by its manufacturer, is not guaranteed or endorsed by the publisher.
